# Supplementary figures and images for: Transcriptomic analysis of a wild and a cultivated varieties of Capsicum annuum over fruit development and ripening
Source: PLoS One. 2021 Aug 24;16(8):e0256319. doi: 10.1371/journal.pone.0256319 (PMC8384167; doi:10.1371/journal.pone.0256319)

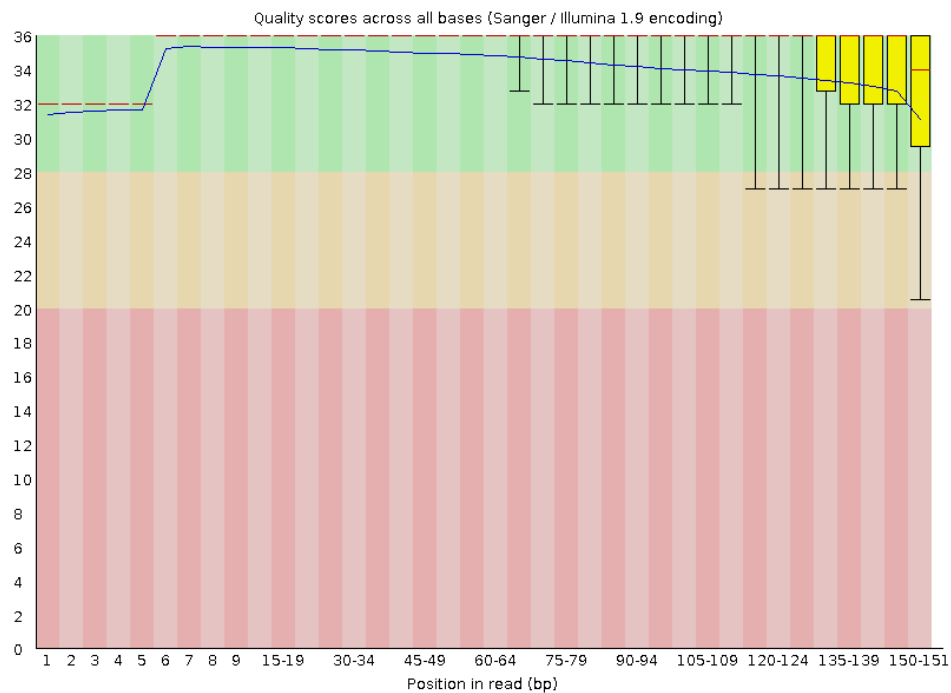

(a)

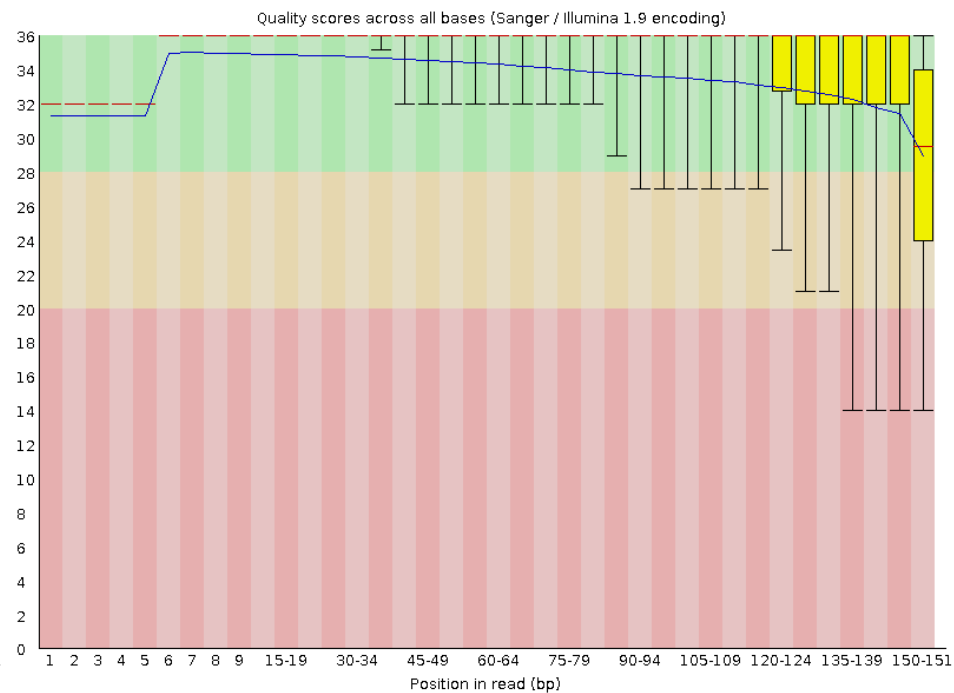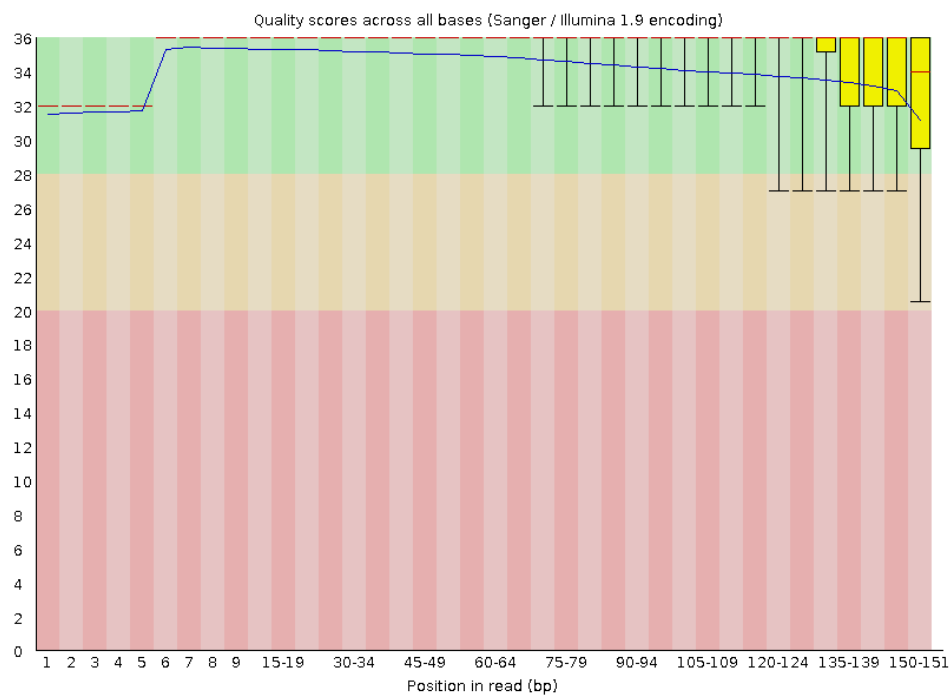

(b)

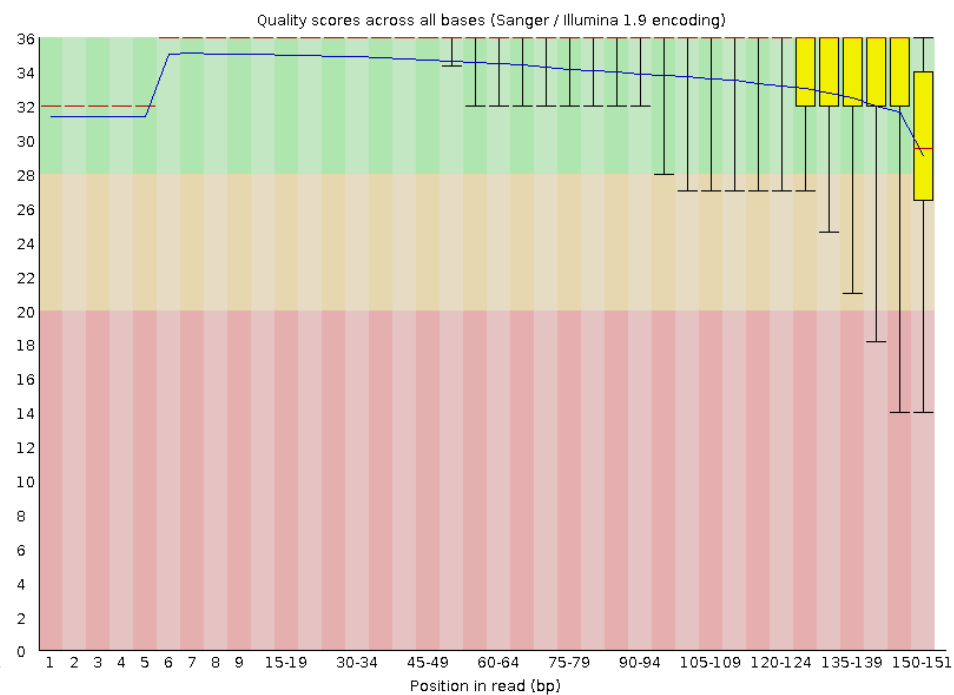

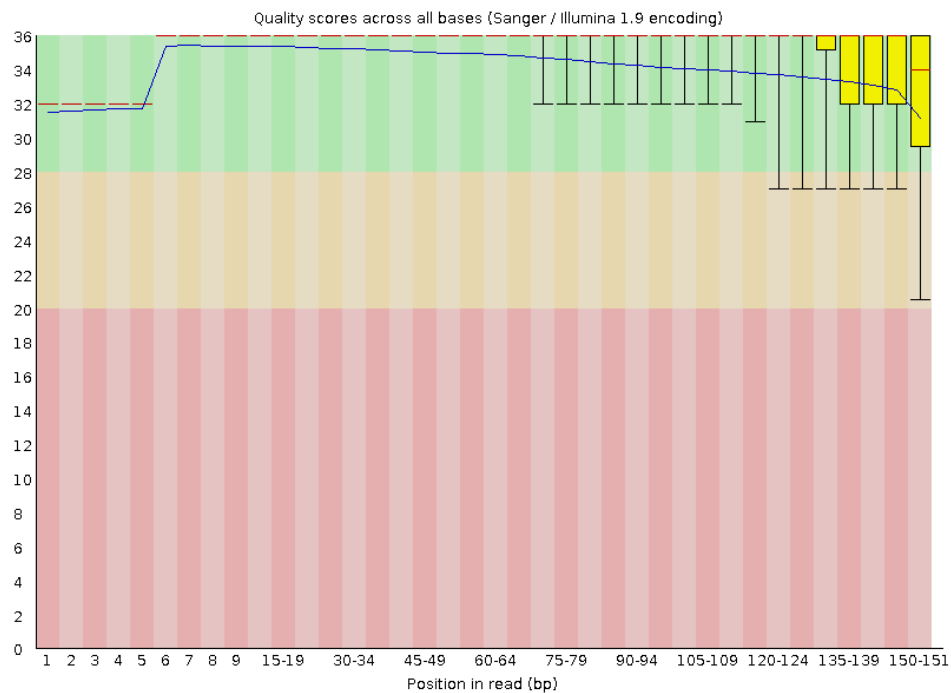

(c)

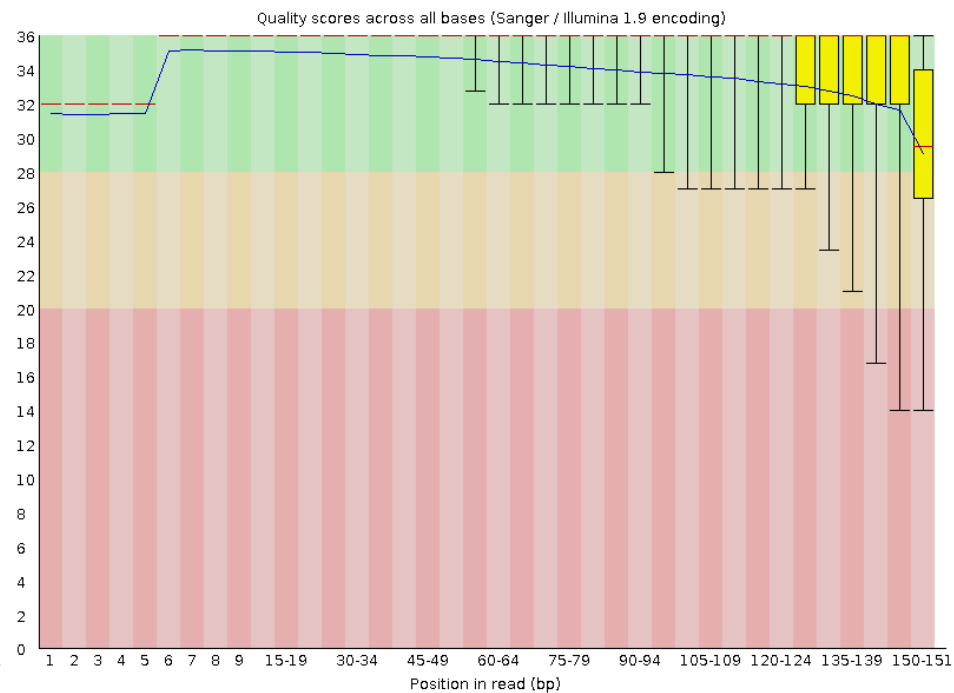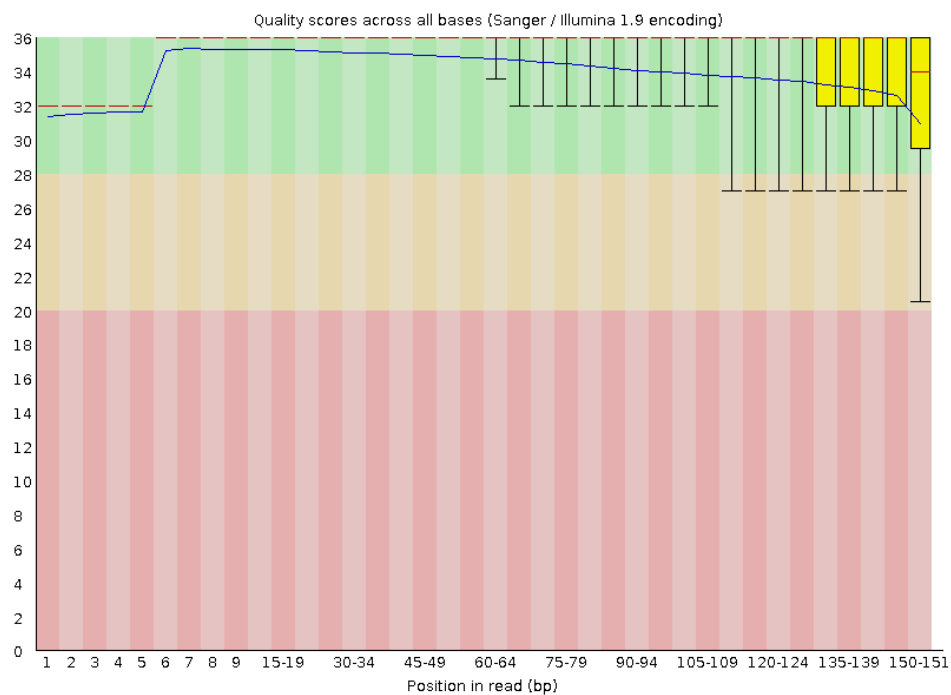

(d)

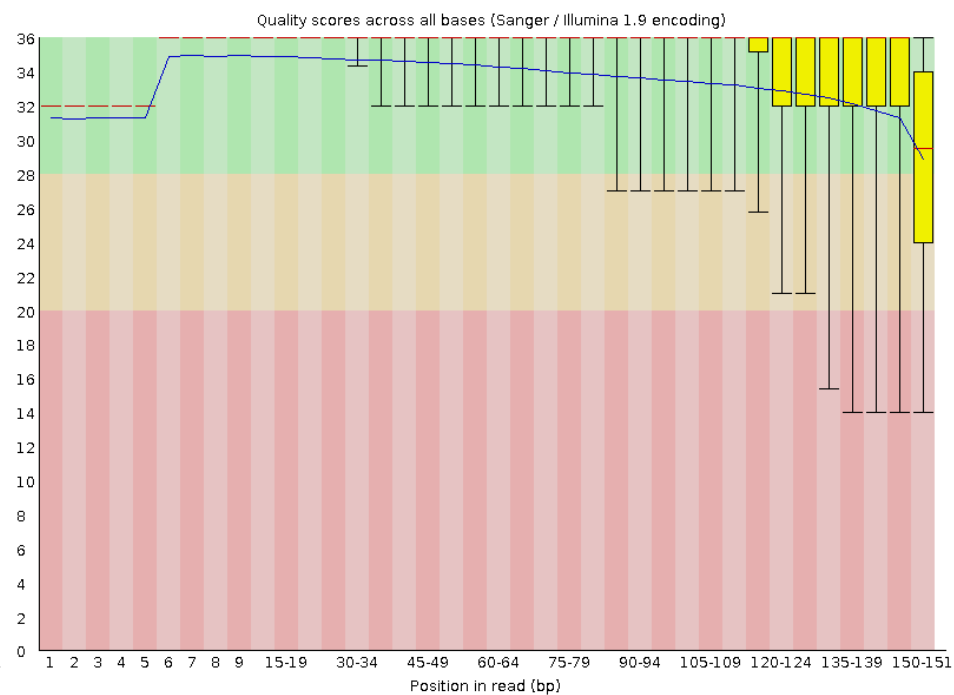

Supplement: S1 Fig — Per base quality of raw Chiltepin RNA-Seq lectures: A) 20 DAA replicate 1, B) 20 DAA replicate 2, C) 68 DAA replicate 1, D) 68 DAA replicate 2. In all cases the plot on the left shows the FastQC result for forward raw reads and the plot on the right shows the FastQC results for reverse raw sequencing reads. (PDF) [file pone.0256319.s001.pdf]

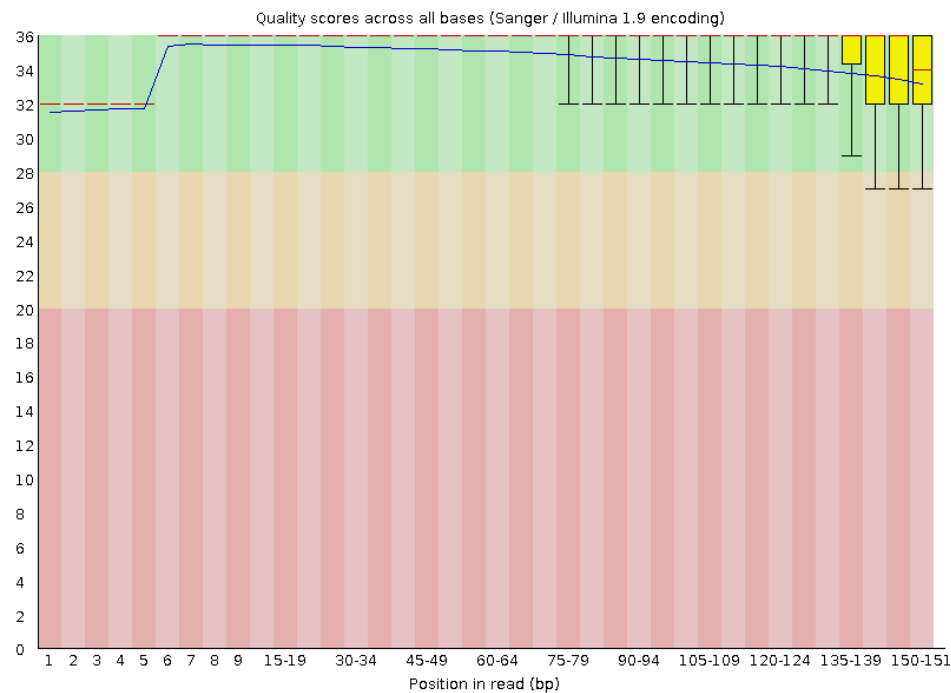

(a)

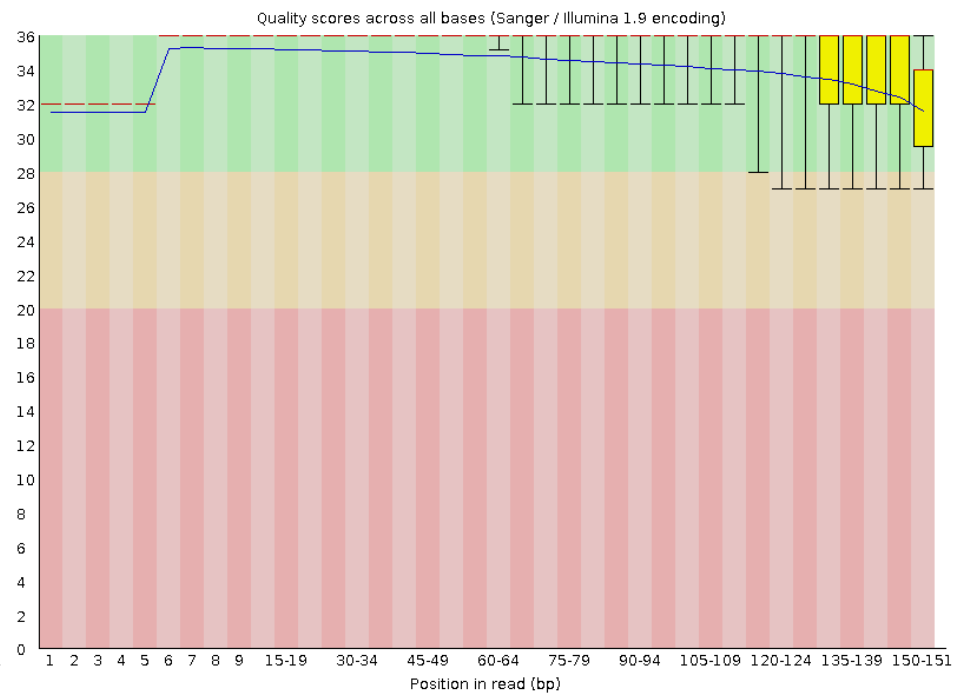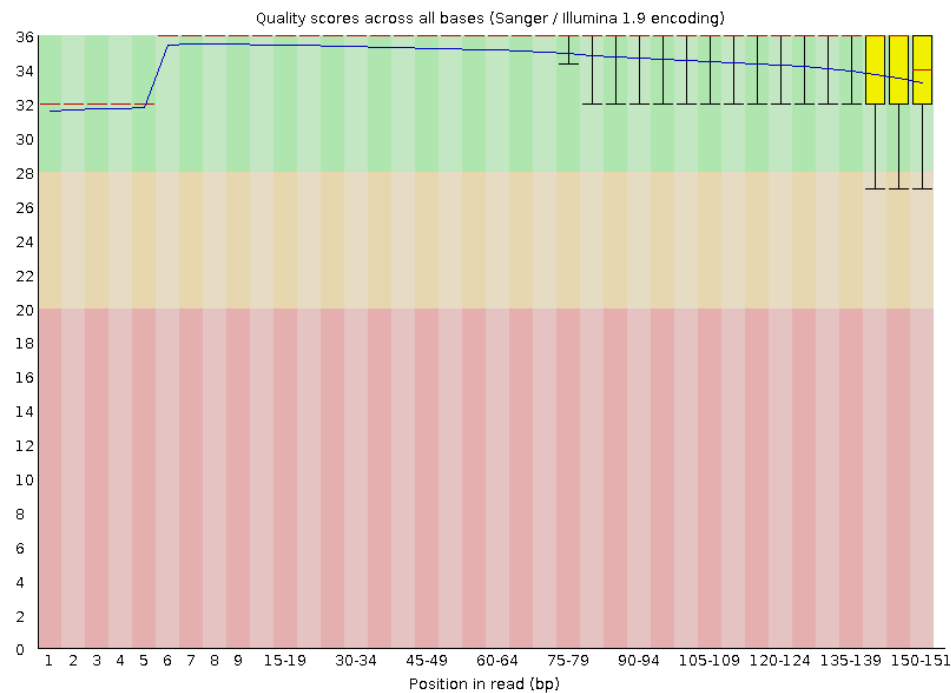

(b)

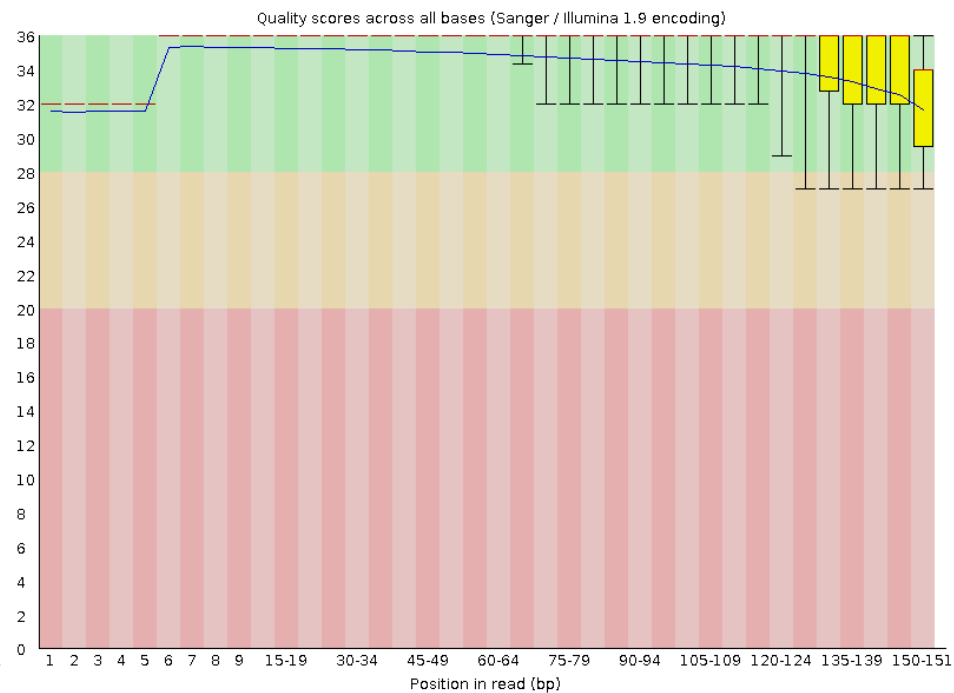

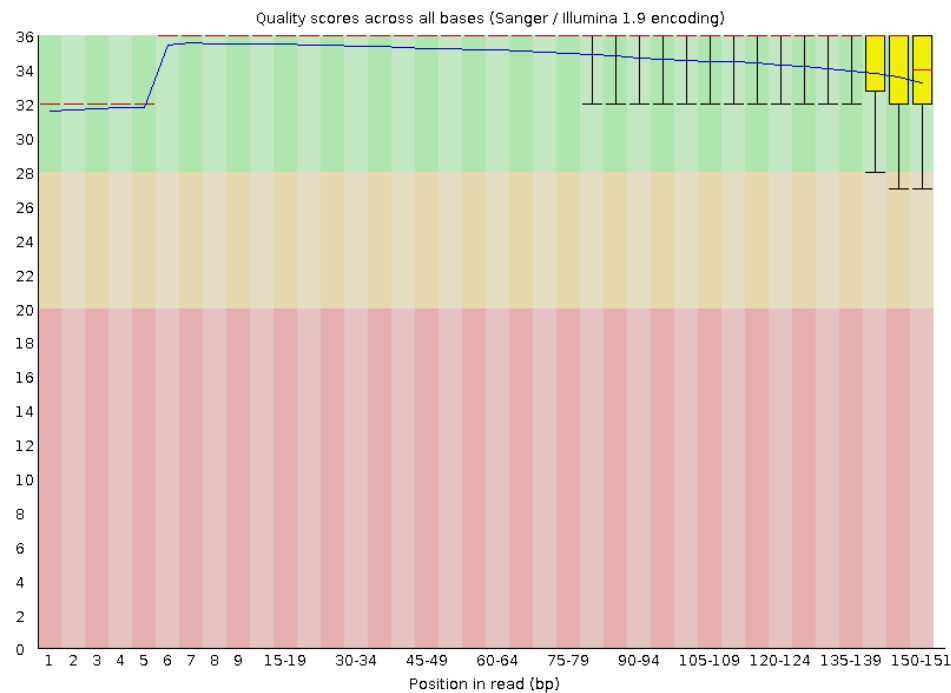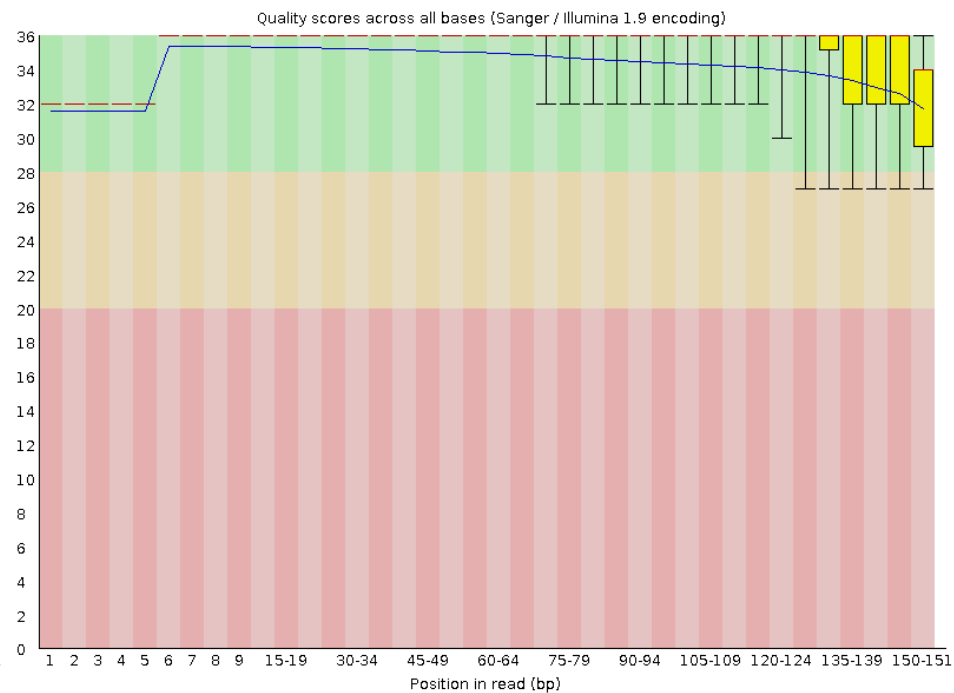

(c)

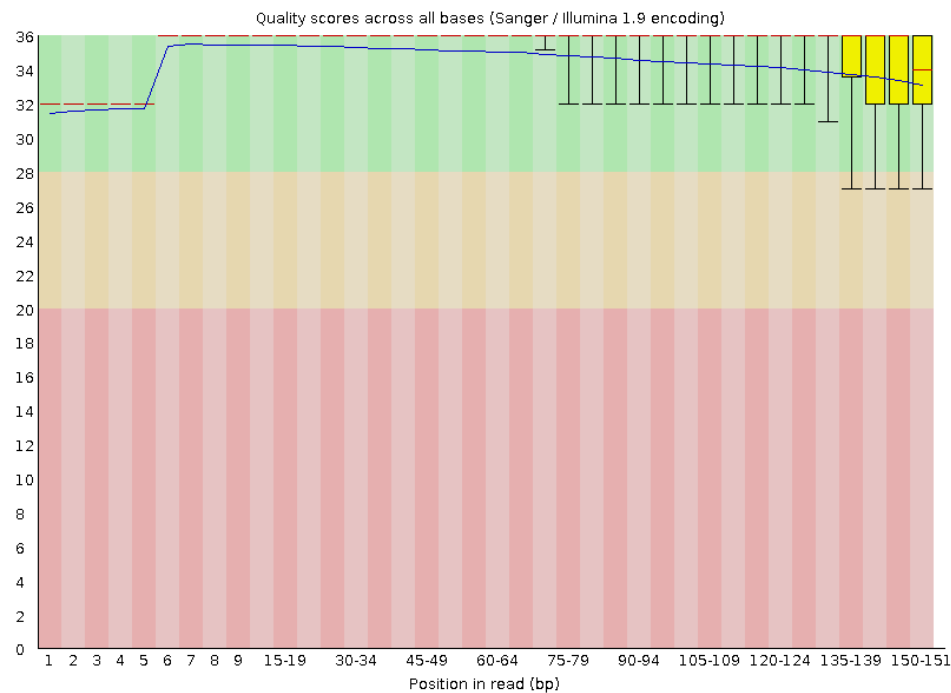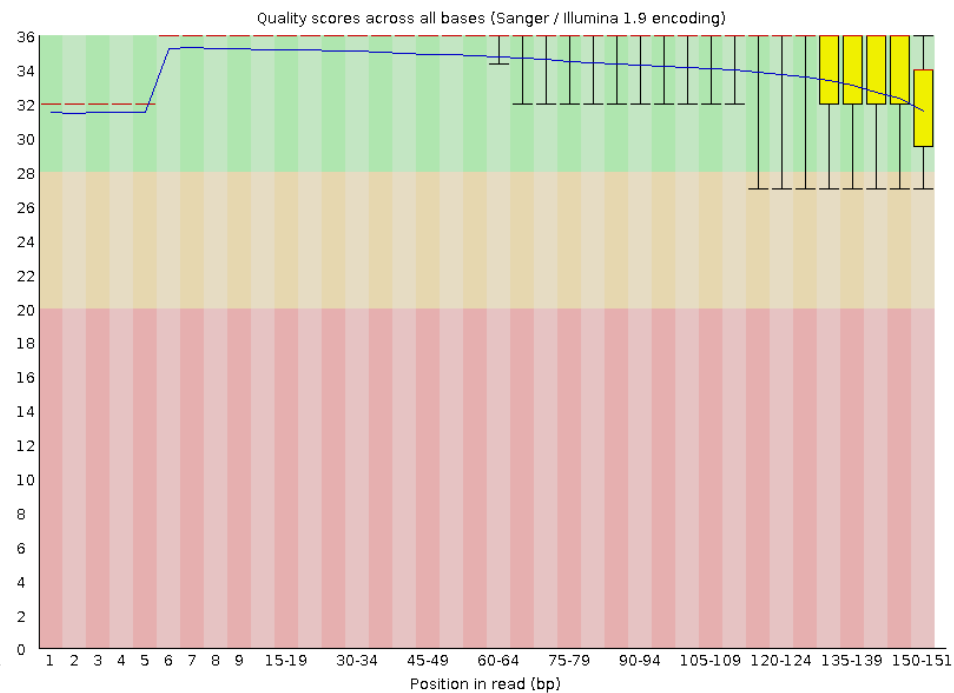

(d)

Supplement: S2 Fig — Per base quality of filtered Chiltepin RNA-Seq lectures: A) 20 DAA replicate 1, B) 20 DAA replicate 2, C) 68 DAA replicate 1, D) 68 DAA replicate 2. In all cases the plot on the left shows the FastQC result for forward trimmed reads and the plot on the right shows the FastQC results for reverse trimmered sequencing reads. (PDF) [file pone.0256319.s002.pdf]

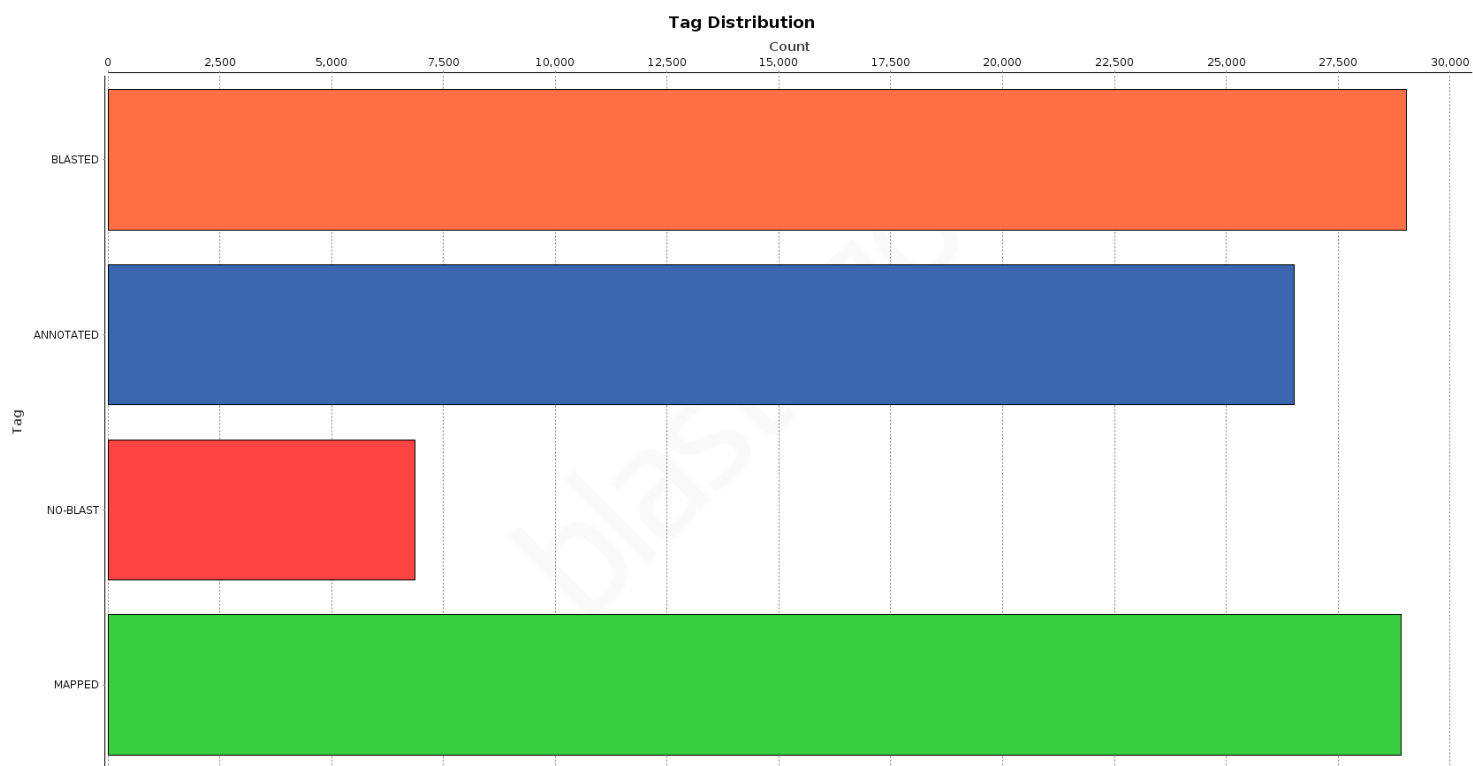

(a)

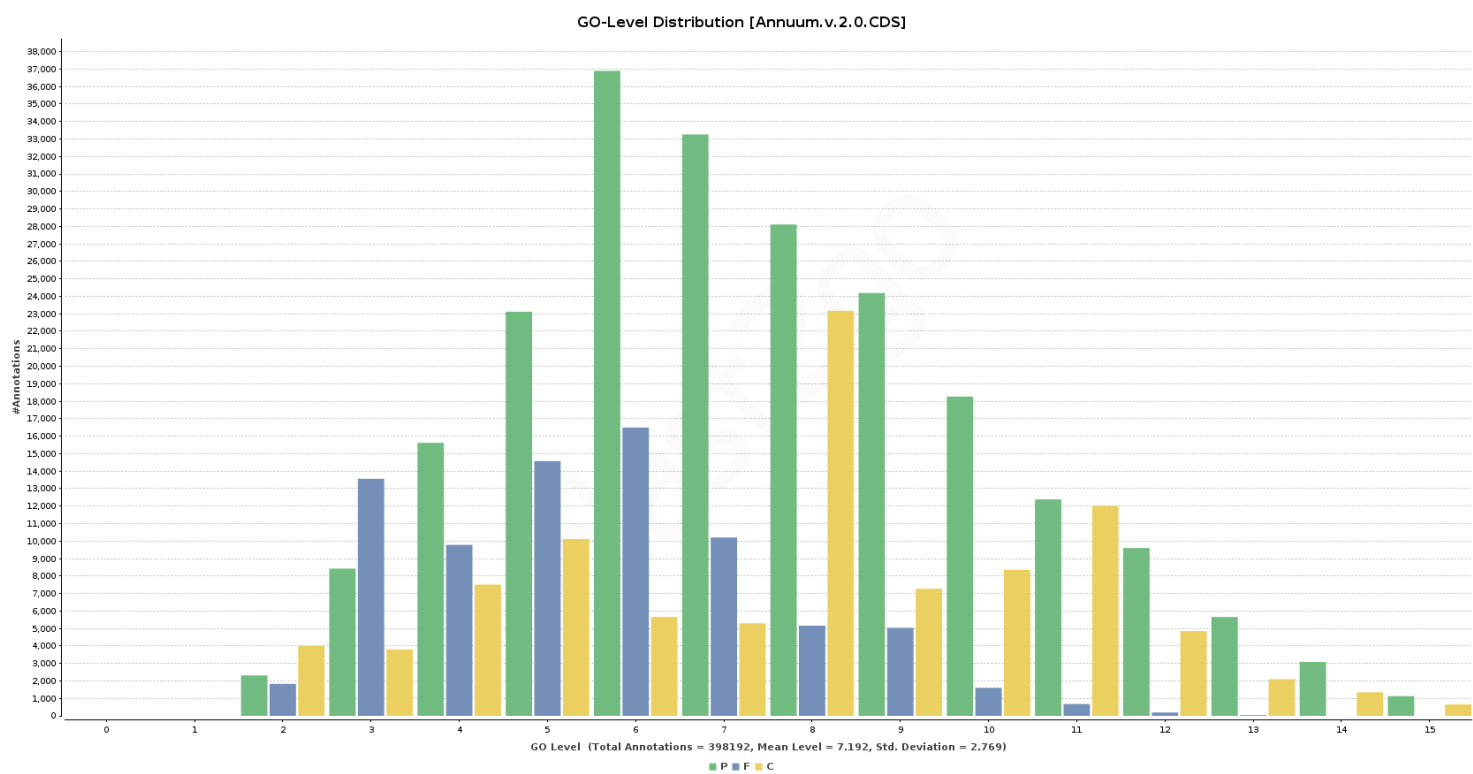

(b)

Supplement: S3 Fig — Statistics of Capsicum annuum L. cv. ‘Criollo de Morelos’ genes annotated via BLAST2GO. A) BLAST2GO gene tag distribution, displays the number of genes with no Blast, blasted, annotated, and mapped. B) BLAST2GO GO level distribution, shows the number of annotations per GO level for biological process, molecular function and cellular component ontologies. (PDF) [file pone.0256319.s003.pdf]

-1.2e+01 1.8e-15 1.2e+01

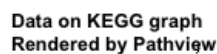

(a)

Supplement: S4 Fig — Metabolic pathway enriched in the contrast Ch20-Ch68, green boxes indicate enzymes encoded by genes repressed, red boxes indicate enzymes encoded by genes induced in the contrast. (PDF) [file pone.0256319.s004.pdf]

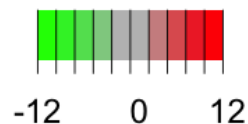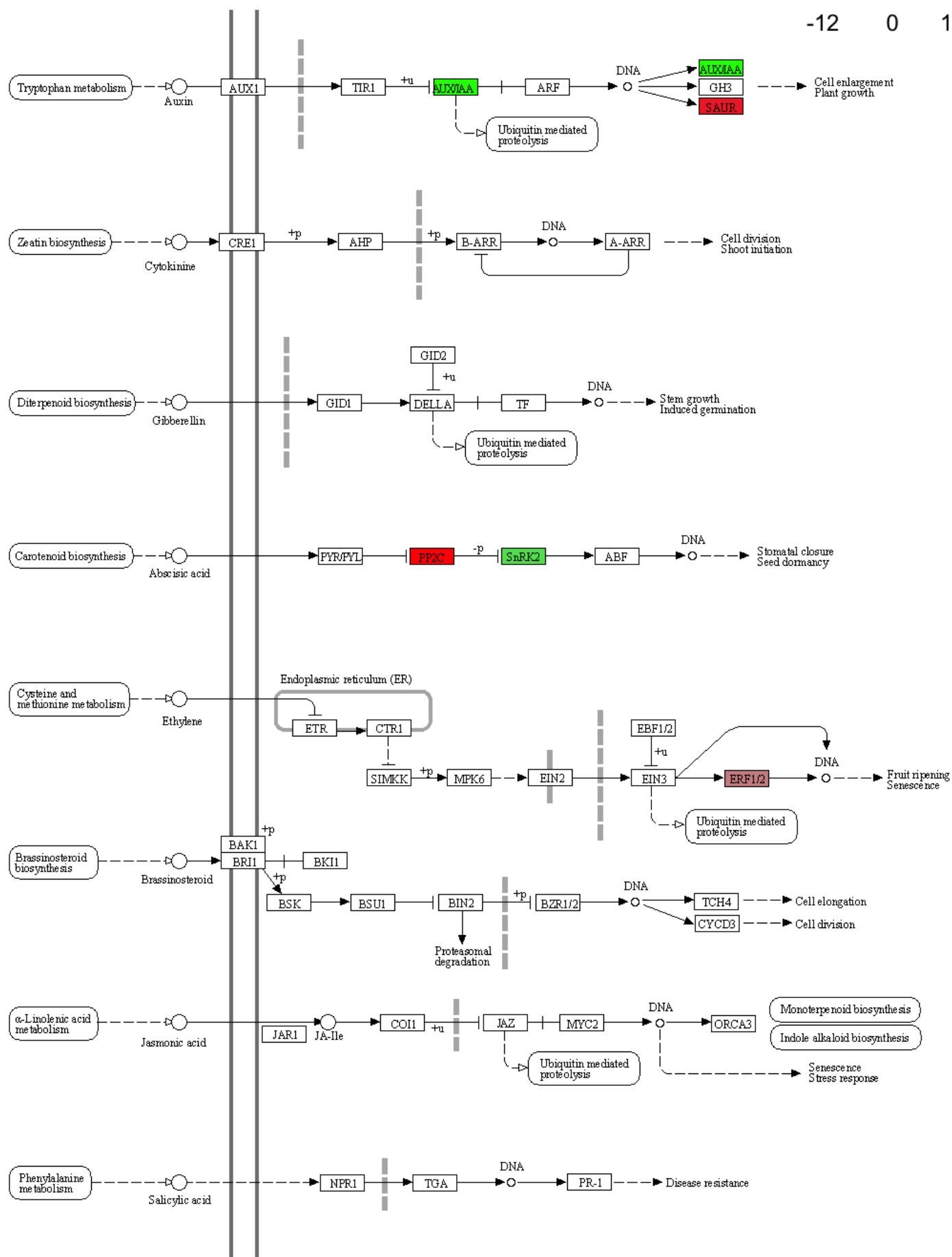

Supplement: S8 Fig — Metabolic pathway enriched in the contrast St20-St60 (Serrano 20 DAA vs Serrano 60 DAA). Green boxes indicate enzymes encoded by genes repressed in the corresponding contrast, red boxes indicate enzymes encoded by genes induced in the corresponding contrast. (PDF) [file pone.0256319.s008.pdf]
