# Supplementary material for: Transcriptomic analysis of a wild and a cultivated varieties of Capsicum annuum over fruit development and ripening
Source: PLoS One. 2021 Aug 24;16(8):e0256319. doi: 10.1371/journal.pone.0256319 (PMC8384167; doi:10.1371/journal.pone.0256319)
Supplement: S5 Fig — Metabolic pathway enriched in the contrast: A) St20-St60 (Serrano 20 DAA vs Serrano 60 DAA), B) Ch20-Ch68 (Chiltepin 20 DAA vs Chiltepin 68 DAA). Green boxes indicate enzymes encoded by genes repressed in the corresponding contrast, red boxes indicate enzymes encoded by genes induced in the corresponding contrast. (PDF) [file pone.0256319.s005.pdf]

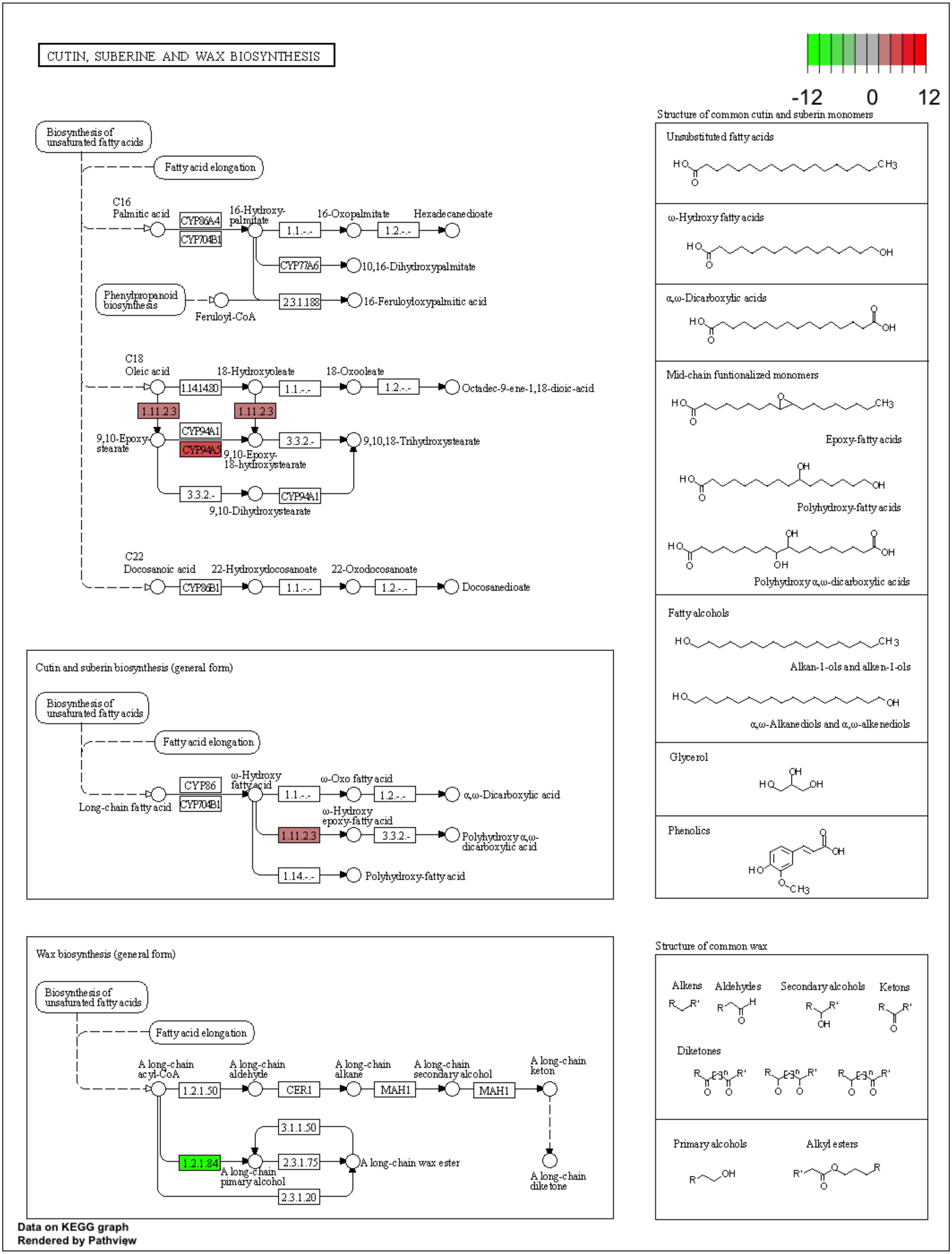
CUTIN, SUBERINE AND WAX BIOSYNTHESIS
-12
0
12
Structure of common cutin and suberin monomers
Unsubstituted fatty acids
ω-Hydroxy fatty acids
α,ω-Dicarboxylic acids
Mid-chain funtionalized monomers
Epoxy-fatty acids
Polyhydroxy-fatty acids
Polyhydroxy α,ω-dicarboxylic acids
Fatty alcohols
Alkan-1-ols and alken-1-ols
α,ω-Alkanediols and α,ω-alkenediols
Glycerol
Phenolics
Biosynthesis of unsaturated fatty acids
Fatty acid elongation
C16 Palmitic acid
CYP86A4
CYP704B1
16-Hydroxy-palmitate
1.1.-.-
16-Oxopalmitate
1.2.-.-
Hexadecanedioate
CYP77A6
10,16-Dihydroxypalmitate
Phenylpropanoid biosynthesis
Feruloyl-CoA
2.3.1.188
16-Feruloyloxypalmitic acid
C18 Oleic acid
1.14.14.80
18-Hydroxyoleate
18-Oxooleate
Octadec-9-ene-1,18-dioic-acid
1.11.2.3
9,10-Epoxy-stearate
CYP94A1
CYP94A5
9,10-Epoxy-18-hydroxystearate
3.3.2.-
9,10,18-Trihydroxystearate
9,10-Dihydroxystearate
C22 Docosanoic acid
CYP86B1
22-Hydroxydocosanoate
22-Oxodocosanoate
Docosanedioate
Cutin and suberin biosynthesis (general form)
CYP86
Long-chain fatty acid
ω-Hydroxy fatty acid
ω-Oxo fatty acid
α,ω-Dicarboxylic acid
ω-Hydroxy epoxy-fatty acid
Polyhydroxy α,ω-dicarboxylic acid
1.14.-.-
Polyhydroxy-fatty acid
Wax biosynthesis (general form)
A long-chain acyl-CoA
1.2.1.50
A long-chain aldehyde
CER1
A long-chain alkane
MAH1
A long-chain secondary alcohol
A long-chain keton
A long-chain diketone
3.1.1.50
1.2.1.84
2.3.1.75
A long-chain pimary alcohol
A long-chain wax ester
2.3.1.20
Structure of common wax
Alkens
Aldehydes
Secondary alcohols
Ketons
Diketones
Primary alcohols
Alkyl esters
Data on KEGG graph
Rendered by Pathview

(a)

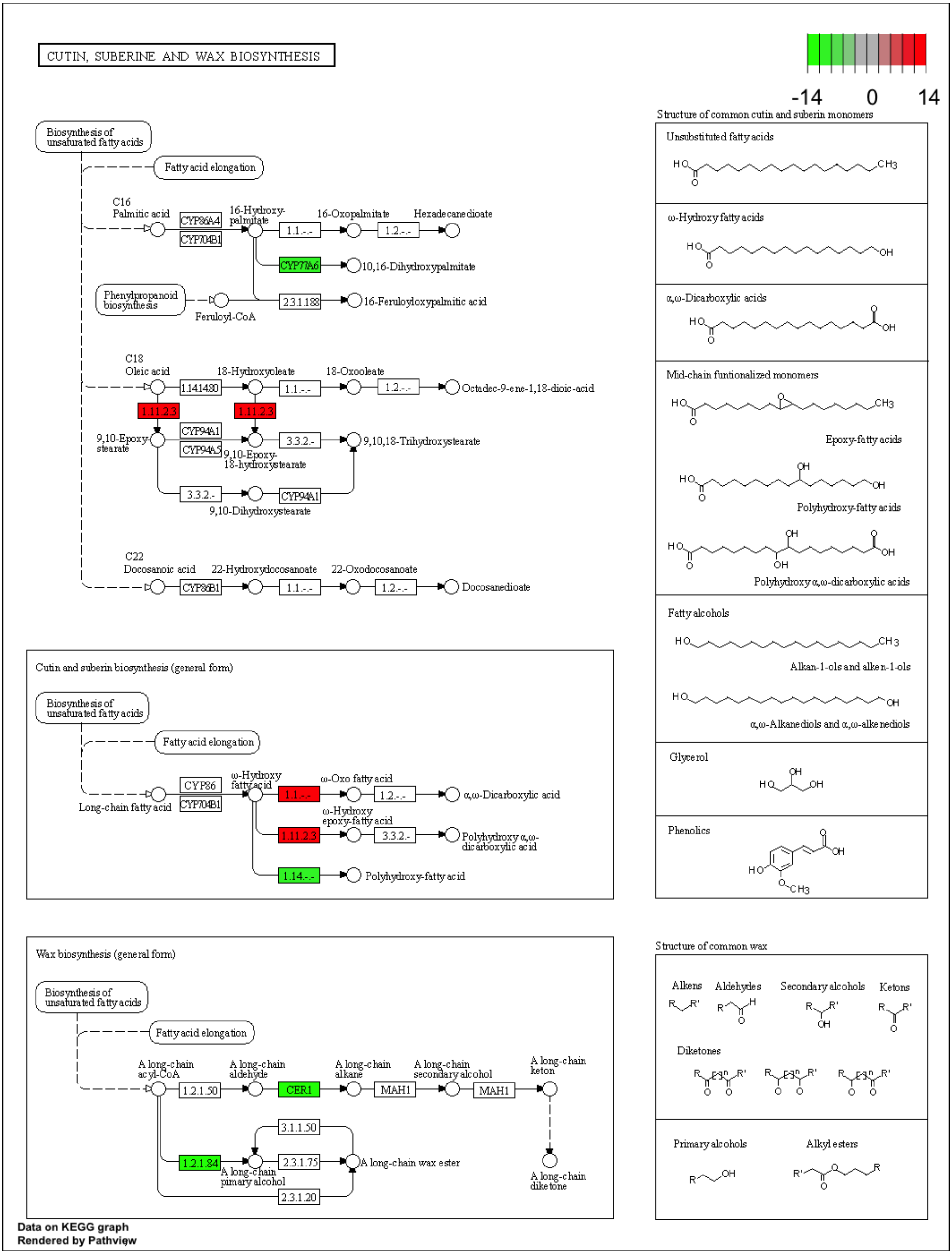
CUTIN, SUBERINE AND WAX BIOSYNTHESIS
-14 0 14
Structure of common cutin and suberin monomers
Unsubstituted fatty acids
ω-Hydroxy fatty acids
α,ω-Dicarboxylic acids
Mid-chain funtionalized monomers
Epoxy-fatty acids
Polyhydroxy-fatty acids
Polyhydroxy α,ω-dicarboxylic acids
Fatty alcohols
Alkan-1-ols and alken-1-ols
α,ω-Alkanediols and α,ω-alkenediols
Glycerol
Phenolics
Biosynthesis of unsaturated fatty acids
Fatty acid elongation
C16 Palmitic acid
CYP86A4
CYP704B1
16-Hydroxy-palmitate
1.1.-.-
16-Oxopalmitate
1.2.-.-
Hexadecanedioate
CYP77A6
10,16-Dihydroxypalmitate
Phenylpropanoid biosynthesis
Feruloyl-CoA
2.3.1.188
16-Feruloyloxypalmitic acid
C18 Oleic acid
1.14.14.80
18-Hydroxyoleate
18-Oxooleate
Octadec-9-ene-1,18-dioic-acid
1.11.2.3
9,10-Epoxy-stearate
CYP94A1
CYP94A5
9,10-Epoxy-18-hydroxystearate
3.3.2.-
9,10,18-Trihydroxystearate
9,10-Dihydroxystearate
C22 Docosanoic acid
CYP86B1
22-Hydroxydocosanoate
22-Oxodocosanoate
Docosanedioate
Cutin and suberin biosynthesis (general form)
Long-chain fatty acid
CYP86
ω-Hydroxy fatty acid
ω-Oxo fatty acid
α,ω-Dicarboxylic acid
ω-Hydroxy epoxy-fatty acid
Polyhydroxy α,ω-dicarboxylic acid
1.14.-.-
Polyhydroxy-fatty acid
Wax biosynthesis (general form)
A long-chain acyl-CoA
1.2.1.50
A long-chain aldehyde
CER1
A long-chain alkane
MAH1
A long-chain secondary alcohol
A long-chain keton
A long-chain diketone
1.2.1.84
A long-chain pimary alcohol
3.1.1.50
2.3.1.75
2.3.1.20
A long-chain wax ester
Structure of common wax
Alkens
Aldehydes
Secondary alcohols
Ketons
Diketones
Primary alcohols
Alkyl esters
Data on KEGG graph
Rendered by Pathview

(b)
