# Supplementary material for: Transcriptomic analysis of a wild and a cultivated varieties of Capsicum annuum over fruit development and ripening
Source: PLoS One. 2021 Aug 24;16(8):e0256319. doi: 10.1371/journal.pone.0256319 (PMC8384167; doi:10.1371/journal.pone.0256319)
Supplement: S6 Fig — Metabolic pathway enriched in the contrast: A) St20-St60 (Serrano 20 DAA vs Serrano 60 DAA), B) Ch20-Ch68 (Chiltepin 20 DAA vs Chiltepin 68 DAA). Green boxes indicate enzymes encoded by genes repressed in the corresponding contrast, red boxes indicate enzymes encoded by genes induced in the corresponding contrast. (PDF) [file pone.0256319.s006.pdf]

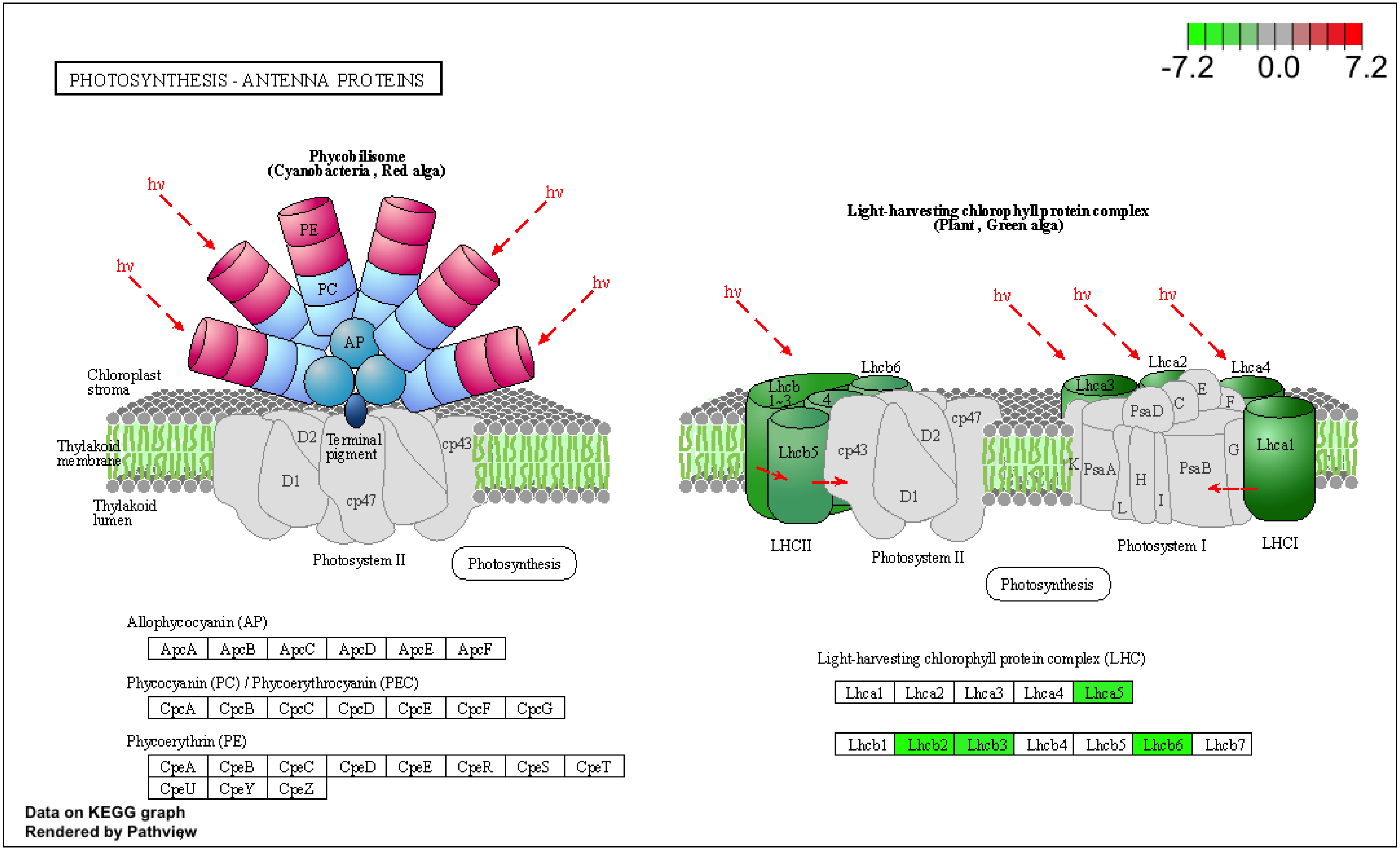
-7.2
0.0
7.2
PHOTOSYNTHESIS - ANTENNA PROTEINS
Phycobilisome
(Cyanobacteria , Red alga)
hν
PE
PC
AP
Chloroplast stroma
Thylakoid membrane
Thylakoid lumen
D2
Terminal pigment
cp43
D1
cp47
Photosystem II
Photosynthesis
Light-harvesting chlorophyll protein complex
(Plant , Green alga)
Lhcb6
Lhcb 1~3
4
Lhcb5
cp43
D2
cp47
D1
Lhca3
Lhca2
Lhca4
PsaD
C
E
F
K
PsaA
H
L
I
PsaB
G
Lhca1
LHCII
Photosystem II
Photosystem I
LHCI
Photosynthesis
Allophycocyanin (AP)
ApcA
ApcB
ApcC
ApcD
ApcE
ApcF
Phycocyanin (PC) / Phycoerythrocyanin (PEC)
CpcA
CpcB
CpcC
CpcD
CpcE
CpcF
CpcG
Phycoerythrin (PE)
CpeA
CpeB
CpeC
CpeD
CpeE
CpeR
CpeS
CpeT
CpeU
CpeY
CpeZ
Light-harvesting chlorophyll protein complex (LHC)
Lhca1
Lhca2
Lhca3
Lhca4
Lhca5
Lhcb1
Lhcb2
Lhcb3
Lhcb4
Lhcb5
Lhcb6
Lhcb7
Data on KEGG graph
Rendered by Pathview

(a)

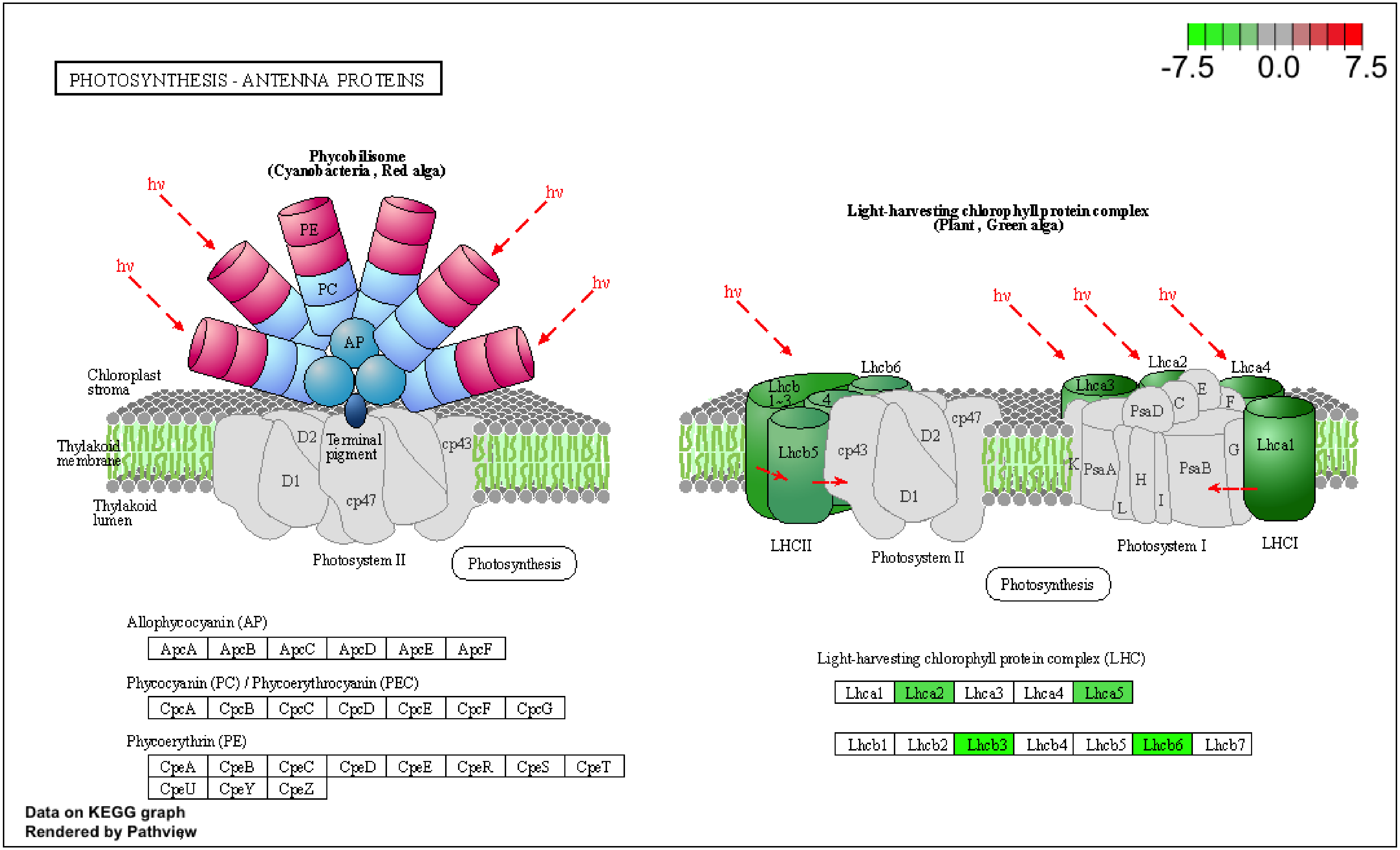
PHOTOSYNTHESIS - ANTENNA PROTEINS
-7.5
0.0
7.5
Phycobilisome
(Cyanobacteria, Red alga)
hv
PE
PC
AP
Chloroplast stroma
Thylakoid membrane
Thylakoid lumen
D2
Terminal pigment
cp43
D1
cp47
Photosystem II
Photosynthesis
Light-harvesting chlorophyll protein complex
(Plant, Green alga)
Lhcb6
Lhcb 1~3
4
Lhcb5
cp43
D2
cp47
D1
Lhca3
Lhca2
Lhca4
PsaD
C
E
F
K
PsaA
H
L
I
PsaB
G
Lhca1
LHCII
Photosystem II
Photosystem I
LHCI
Photosynthesis
Allophycocyanin (AP)
ApcA
ApcB
ApcC
ApcD
ApcE
ApcF
Phycocyanin (PC) / Phycoerythrocyanin (PEC)
CpcA
CpcB
CpcC
CpcD
CpcE
CpcF
CpcG
Phycoerythrin (PE)
CpeA
CpeB
CpeC
CpeD
CpeE
CpeR
CpeS
CpeT
CpeU
CpeY
CpeZ
Light-harvesting chlorophyll protein complex (LHC)
Lhca1
Lhca2
Lhca3
Lhca4
Lhca5
Lhcb1
Lhcb2
Lhcb3
Lhcb4
Lhcb5
Lhcb6
Lhcb7
Data on KEGG graph
Rendered by Pathview

(b)
