# Supplementary material for: Transcriptomic analysis of a wild and a cultivated varieties of Capsicum annuum over fruit development and ripening
Source: PLoS One. 2021 Aug 24;16(8):e0256319. doi: 10.1371/journal.pone.0256319 (PMC8384167; doi:10.1371/journal.pone.0256319)
Supplement: S9 Fig — Metabolic pathway enriched in the contrast Ch20-Ch68 (Chiltepin 20 DAA vs Chiltepin 68 DAA). Green boxes indicate enzymes encoded by genes repressed in the corresponding contrast, red boxes indicate enzymes encoded by genes induced in the corresponding contrast. (PDF) [file pone.0256319.s009.pdf]

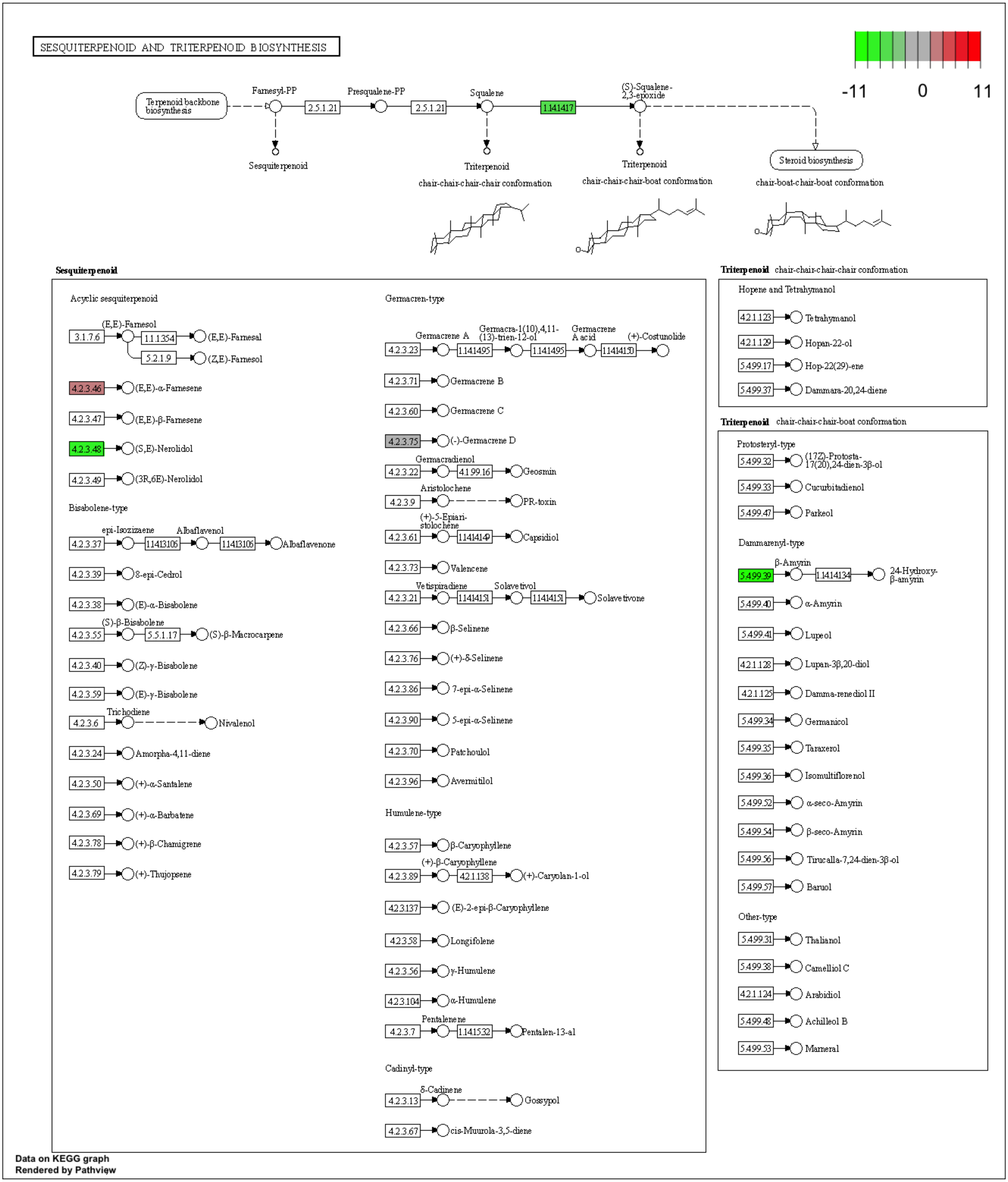
SESQUITERPENOID AND TRITERPENOID BIOSYNTHESIS
-11
0
11
Terpenoid backbone biosynthesis
Farnesyl-PP
2.5.1.21
Presqualene-PP
2.5.1.21
Squalene
1.14.14.17
(S)-Squalene-2,3-epoxide
Sesquiterpenoid
Triterpenoid
chair-chair-chair-chair conformation
Triterpenoid
chair-chair-chair-boat conformation
Steroid biosynthesis
chair-boat-chair-boat conformation
Sesquiterpenoid
Acyclic sesquiterpenoid
(E,E)-Farnesol
3.1.7.6
1.1.1.354
(E,E)-Farnesal
5.2.1.9
(Z,E)-Farnesol
4.2.3.46
(E,E)-α-Farnesene
4.2.3.47
(E,E)-β-Farnesene
4.2.3.48
(S,E)-Nerolidol
4.2.3.49
(3R,6E)-Nerolidol
Bisabolene-type
epi-Isozizaene
Albaflavenol
4.2.3.37
1.14.13.106
1.14.13.106
Albaflavenone
4.2.3.39
8-epi-Cedrol
4.2.3.38
(E)-α-Bisabolene
(S)-β-Bisabolene
4.2.3.55
5.5.1.17
(S)-β-Macrocarpene
4.2.3.40
(Z)-γ-Bisabolene
4.2.3.59
(E)-γ-Bisabolene
Trichodiene
4.2.3.6
Nivalenol
4.2.3.24
Amorpha-4,11-diene
4.2.3.50
(+)-α-Santalene
4.2.3.69
(+)-α-Barbatene
4.2.3.78
(+)-β-Chamigrene
4.2.3.79
(+)-Thujopsene
Germacren-type
Germacrene A
Germacra-1(10),4,11-(13)-trien-12-ol
Germacrene A acid
(+)-Costunolide
4.2.3.23
1.14.14.95
1.14.14.95
1.14.14.150
4.2.3.71
Germacrene B
4.2.3.60
Germacrene C
4.2.3.75
(-)-Germacrene D
Germacradienol
4.2.3.22
4.1.99.16
Geosmin
Aristolochene
4.2.3.9
PR-toxin
(+)-5-Epiaristolochene
4.2.3.61
1.14.14.149
Capsidiol
4.2.3.73
Valencene
Vetispiradiene
Solavetivol
4.2.3.21
1.14.14.151
1.14.14.151
Solavetivone
4.2.3.66
β-Selinene
4.2.3.76
(+)-δ-Selinene
4.2.3.86
7-epi-α-Selinene
4.2.3.90
5-epi-α-Selinene
4.2.3.70
Patchoulol
4.2.3.96
Avermitilol
Humulene-type
4.2.3.57
β-Caryophyllene
(+)-β-Caryophyllene
4.2.3.89
4.2.1.138
(+)-Caryolan-1-ol
4.2.3.137
(E)-2-epi-β-Caryophyllene
4.2.3.58
Longifolene
4.2.3.56
γ-Humulene
4.2.3.104
α-Humulene
Pentalenene
4.2.3.7
1.14.15.32
Pentalen-13-al
Cadinyl-type
δ-Cadinene
4.2.3.13
Gossypol
4.2.3.67
cis-Muurola-3,5-diene
Triterpenoid chair-chair-chair-chair conformation
Hopene and Tetrahymanol
4.2.1.123
Tetrahymanol
4.2.1.129
Hopan-22-ol
5.4.99.17
Hop-22(29)-ene
5.4.99.37
Dammara-20,24-diene
Triterpenoid chair-chair-chair-boat conformation
Protosteryl-type
5.4.99.32
(17Z)-Protosta-17(20),24-dien-3β-ol
5.4.99.33
Cucurbitadienol
5.4.99.47
Parkeol
Dammarenyl-type
β-Amyrin
5.4.99.39
1.14.14.134
24-Hydroxy-β-amyrin
5.4.99.40
α-Amyrin
5.4.99.41
Lupeol
4.2.1.128
Lupan-3β,20-diol
4.2.1.125
Dammarenediol II
5.4.99.34
Germanicol
5.4.99.35
Taraxerol
5.4.99.36
Isomultiflorenol
5.4.99.52
α-seco-Amyrin
5.4.99.54
β-seco-Amyrin
5.4.99.56
Tirucalla-7,24-dien-3β-ol
5.4.99.57
Baruol
Other-type
5.4.99.31
Thalianol
5.4.99.38
Camelliol C
4.2.1.124
Arabidiol
5.4.99.48
Achilleol B
5.4.99.53
Marneral
Data on KEGG graph
Rendered by Pathview
