# Supplementary material for: Transcriptomic analysis of a wild and a cultivated varieties of Capsicum annuum over fruit development and ripening
Source: PLoS One. 2021 Aug 24;16(8):e0256319. doi: 10.1371/journal.pone.0256319 (PMC8384167; doi:10.1371/journal.pone.0256319)
Supplement: S7 Fig — Metabolic pathway enriched in the contrast St20-St60 (Serrano 20 DAA vs Serrano 60 DAA). Green boxes indicate enzymes encoded by genes repressed in the corresponding contrast, red boxes indicate enzymes encoded by genes induced in the corresponding contrast. (PDF) [file pone.0256319.s007.pdf]

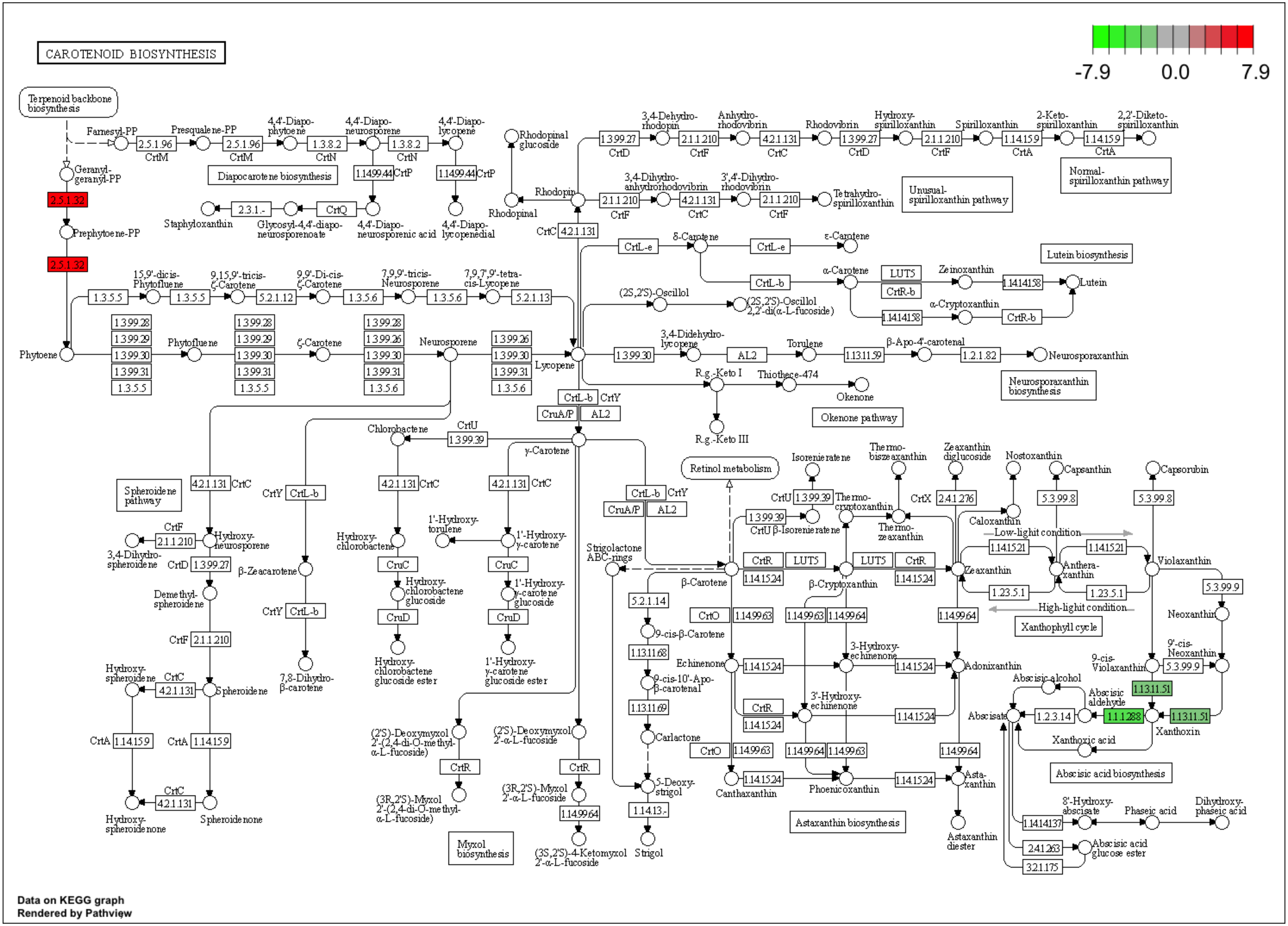

CAROTENOID BIOSYNTHESIS
-7.9
0.0
7.9
Terpenoid backbone biosynthesis
Farnesyl-PP
2.5.1.96
CrtM
Presqualene-PP
4,4'-Diapo-phytoene
1.3.8.2
CrtN
4,4'-Diapo-neurosporene
4,4'-Diapo-lycopene
Diapocarotene biosynthesis
1.14.99.44
CrtP
2.3.1.-
CrtQ
Staphyloxanthin
Glycosyl-4,4'-diapo-neurosporenoate
4,4'-Diapo-neurosporenic acid
4,4'-Diapo-lycopenedial
Geranyl-geranyl-PP
2.5.1.32
Prephytoene-PP
Rhodopinal glucoside
Rhodopinal
Rhodopin
CrtC
4.2.1.131
1.3.99.27
CrtD
3,4-Dehydro-rhodopin
2.1.1.210
CrtF
Anhydro-rhodovibrin
CrtC
Rhodovibrin
Hydroxy-spirilloxanthin
Spirilloxanthin
1.14.15.9
CrtA
2-Keto-spirilloxanthin
2,2'-Diketo-spirilloxanthin
Normal-spirilloxanthin pathway
3,4-Dihydro-anhydrorhodovibrin
3',4'-Dihydro-rhodovibrin
Tetrahydro-spirilloxanthin
Unusual-spirilloxanthin pathway
CrtL-e
δ-Carotene
ε-Carotene
Lutein biosynthesis
CrtL-b
α-Carotene
LUT5
CrtR-b
Zeinoxanthin
1.14.14.158
Lutein
α-Cryptoxanthin
(2S,2'S)-Oscillol
(2S,2'S)-Oscillol 2,2'-di(α-L-fucoside)
15,9'-dicis-Phytofluene
9,15,9'-tricis-ζ-Carotene
9,9'-Di-cis-ζ-Carotene
7,9,9'-tricis-Neurosporene
7,9,7',9'-tetra-cis-Lycopene
1.3.5.5
5.2.1.12
1.3.5.6
5.2.1.13
1.3.99.28
1.3.99.29
1.3.99.30
1.3.99.31
1.3.99.26
Phytoene
Phytofluene
ζ-Carotene
Neurosporene
Lycopene
3,4-Didehydro-lycopene
AL2
Torulene
1.13.11.59
β-Apo-4'-carotenal
1.2.1.82
Neurosporaxanthin
Neurosporaxanthin biosynthesis
R.g.-Keto I
Thiothece-474
Okenone
Okenone pathway
R.g.-Keto III
CrtY
CruA/P
Chlorobactene
CrtU
1.3.99.39
γ-Carotene
Retinol metabolism
Spheroidene pathway
CrtC
Hydroxy-neurosporene
3,4-Dihydro-spheroidene
CrtD
1.3.99.27
β-Zeacarotene
Demethyl-spheroidene
Hydroxy-spheroidene
Spheroidene
Hydroxy-spheroidenone
Spheroidenone
7,8-Dihydro-β-carotene
Hydroxy-chlorobactene
CruC
Hydroxy-chlorobactene glucoside
CruD
Hydroxy-chlorobactene glucoside ester
1'-Hydroxy-torulene
1'-Hydroxy-γ-carotene
1'-Hydroxy-γ-carotene glucoside
1'-Hydroxy-γ-carotene glucoside ester
Strigolactone ABC-rings
Isorenieratene
β-Isorenieratene
Thermo-biszeaxanthin
Thermo-cryptoxanthin
Thermo-zeaxanthin
Zeaxanthin diglucoside
CrtX
2.4.1.276
Nostoxanthin
Caloxanthin
Capsanthin
Capsorubin
5.3.99.8
Low-light condition
1.14.15.21
Antheraxanthin
Violaxanthin
β-Carotene
1.14.15.24
β-Cryptoxanthin
Zeaxanthin
1.23.5.1
5.3.99.9
High-light condition
Xanthophyll cycle
Neoxanthin
5.2.1.14
CrtO
1.14.99.63
1.14.99.64
9-cis-β-Carotene
1.13.11.68
Echinenone
3-Hydroxy-echinenone
Adonixanthin
9'-cis-Neoxanthin
9-cis-Violaxanthin
9-cis-10'-Apo-β-carotenal
1.13.11.69
3'-Hydroxy-echinenone
Abscisic alcohol
Abscisic aldehyde
1.13.11.51
1.1.1.288
1.2.3.14
Abscisate
Xanthoxin
Xanthoxic acid
Carlactone
5-Deoxy-strigol
1.14.13.-
Strigol
Canthaxanthin
Phoenicoxanthin
Astaxanthin
Astaxanthin biosynthesis
Astaxanthin diester
Abscisic acid biosynthesis
8'-Hydroxy-abscisate
Phaseic acid
Dihydroxy-phaseic acid
1.14.14.137
2.4.1.263
Abscisic acid glucose ester
3.2.1.175
(2'S)-Deoxymyxol 2'-(2,4-di-O-methyl-α-L-fucoside)
(2'S)-Deoxymyxol 2'-α-L-fucoside
CrtR
(3R,2'S)-Myxol 2'-(2,4-di-O-methyl-α-L-fucoside)
(3R,2'S)-Myxol 2'-α-L-fucoside
Myxol biosynthesis
1.14.99.64
(3S,2'S)-4-Ketomyxol 2'-α-L-fucoside
Data on KEGG graph
Rendered by Pathview

(a)
