# Supplementary material for: Transcriptomic analysis of a wild and a cultivated varieties of Capsicum annuum over fruit development and ripening
Source: PLoS One. 2021 Aug 24;16(8):e0256319. doi: 10.1371/journal.pone.0256319 (PMC8384167; doi:10.1371/journal.pone.0256319)
Supplement: Attachment — Submitted filename: ResponseToReviewers.pdf [file pone.0256319.s013.pdf]

Response to Reviewers
Manuscript "**Transcriptomic analysis of a wild and a cultivated varieties of *Capsicum annuum* over fruit development and ripening**" (PONE-D-21-14082).

Dear Dr. Xiang Jia Min,

We thank you and the two reviewers for the time and effort invested in the review of our manuscript. Below we address all the points raised. Those points were taken into account to write the new version of the manuscript

Editor requirements

1. We have ensured that the new version of the manuscript meets PLOS ONE's style requirements.
2. We used PLOS ONE's LaTeX template in the new version of the manuscript.
3. Funding-related text was removed from the Acknowledgments section. **Funding Statement:** This work was supported in part by COFUPRO (A/GTO/RGAG-2014-076-Consorcio de Fundaciones PRODUCE). FR-M acknowledges the Mexican Council of Science and Technology (CONACyT) for supporting with a PhD scholarship (261122) during the development of the project. The funders had no role in study design, data collection and analysis, decision to publish, or preparation of the manuscript.
4. Copyrighted images. Figures S4, S5, S6, S7, S8 and S9 contain copyrighted material (KEGG pathway map images). Table below gives details of those figures.

**Supporting figures that contain copyrighted material.**			
**Figure**	**File**	**KEGG id.**	**Description of KEGG pathway**
S4 Fig.	S4_fig.pdf	map00940	Phenylpropanoid biosynthesis
S5 Fig.	S5_fig.pdf	map00073	Cutin, suberine and wax biosynthesis
S6 Fig.	S6_fig.pdf	map00196	Photosynthesis - antenna proteins
S7 Fig.	S7_fig.pdf	map00906	Carotenoid biosynthesis
S8 Fig.	S8_fig.pdf	map04075	Plant hormone signal transduction
S9 Fig.	S9_fig.pdf	map00909	Sesquiterpenoid and triterpenoid biosynthesis

As instructed by the Editor, we obtained written permission from the copyright holder to publish these figures (file "KEGG Copyright Permission 210739.pdf").

Reviewer #1

1. *"Line 7 I was confused by the sentence".* The line was modified by adding "*which consists of*".
2. *"Lines 104-105 paragraph belongs to Discussion."* Those lines were moved to Discussion.
3. *"Lines 117-120 paragraph belongs to Discussion."* Those lines were moved to Discussion.
4. *"Line 119 need to remove the period."* The line was modified.
5. *"Lines 291-295 paragraph belongs to Discussion."* Those lines were moved to Discussion.
6. *"Lines 353-356 Paragraph needs to be rewritten."* The paragraph was rewritten.

Reviewer #2

1. "*In the Results section, Transcriptomic analysis of the wild Chiltepin (CH; Capsicum annuum var. glabriusculum) and the cultivated Serrano Tampiqueño (ST; Capsicum annuum L. cv. 'Tampiqueño 74') was performed at two stages (Fig 1A), the immature stage at 20 days after anthesis (DAA) for both CH and ST, and at mature state at 60 DAA for ST and 68 DAA for CH [45]. I did not find that the fruit of ST*

in Fig. 1A. Moreover, Please check if the reference [45] is appropriate." **Response:** Fig. 1 was modified. Now panel A shows both accessions, and the DAA at which those were sampled are annotated. Reference [45] was removed.

Aditional changes:

1. Author's name was changed from "A. Corina Hayano-Kanashiro" to "Corina Hayano-Kanashiro".
2. Description of Figure 1 A) was changed to include both accessions over different development and maturation stages.
3. Line 514: reference [107] was added to comply with KEGG copyright conditions.
4. Fig 6 was remade to improve resolution and readability.

Octavio Martínez
Corresponding author

Corina Hayano-Kanashiro
Co-corresponding author
